# Synergistic Effect of *Lactobacillus* Mixtures and *Lagerstroemia speciosa* Leaf Extract in Reducing Obesity in High-Fat Diet-Fed Mice

**DOI:** 10.3390/biology13121047

**Published:** 2024-12-13

**Authors:** Kippeum Lee, Hyeon-Ji Kim, Joo Yun Kim, Jae Jung Shim, Jae Hwan Lee

**Affiliations:** R&BD Center, hy Co., Ltd., 22 Giheungdanji-ro 24 Beon-gil, Giheung-gu, Yongin-si 17086, Republic of Korea; joy4917@hanmail.net (K.L.); hyeonjk@hy.co.kr (H.-J.K.); jjshim@hy.co.kr (J.J.S.)

**Keywords:** *Lactobacillus* HY7601 and KY1032, *Lagerstroemia speciosa* leaf extract, obesity, inguinal fat, epididymal fat, adipogenesis

## Abstract

In this study, we describe the anti-obesity effects of a novel combination of *Lactobacillus* mixture (*Lactobacillus curvatus* HY7601 and *Lactobacillus plantarum* KY1032) and leaf extract of *Lagerstroemia speciosa* (*L. speciosa*) in mice. The administration of the probiotic mixture of HY7601 and KY1032 in combination with the leaf extract of *L. speciosa* significantly attenuated fat tissue formation and body weight gain in mice fed a high-fat diet. The white adipose fat mass, comprising the inguinal and epididymal fat pads, was most effectively reduced when the probiotic mixture and *L. speciosa* leaf extract was orally administered to the mice in combination. This combination also reduced the mRNA expression of adipogenic genes (those encoding CCAAT/enhancer-binding protein alpha, peroxisome proliferator-activated receptor gamma, and fatty acid-binding protein 4) in inguinal and epididymal white adipose tissue depots and the liver. Finally, the combination of reduced blood glucose concentrations regulated the insulin resistance of high-fat diet-fed obese mice. These findings provide insight into the mechanisms underlying the effect of this combination and suggest that using *Lactobacillus* mixture (HY7601 and KY1032) is as safe as microbial monotherapy, but more effective at preventing obesity.

## Simple Summary

Obesity is caused by an imbalance between food consumption and energy expenditure. It is a chronic, long-term condition that involves excessive fat accumulation, which can damage health. Fat tissue contributes significantly to metabolism and is also closely associated with resistance to the effects of the insulin hormone. Mammals have the ability to store excess energy in fat cells in the form of triglycerides, which are stored as fat droplets. A mixture of *Lactobacillus* bacteria is a probiotic preparation that has been shown to help people lose weight in previous studies. In the present study, we tested whether a specific *Lactobacillus* mixture would prevent obesity in animals when used in combination with a specific plant extract. We found that a 1:1 combination of *Lagerstroemia* leaf extract and the *Lactobacillus* mixture inhibited the weight gain and expansion of fat tissue in mice that had been made obese by being fed a high-fat diet.

## 1. Introduction

Obesity is closely associated with the development of type 2 diabetes mellitus, non-alcoholic fatty liver, hypertension, cardiovascular disorders, a variety of cancers, and metabolic syndrome [1,2]. It is a metabolic disorder that is caused by the excessive accumulation of body fat [3,4], and it is rapidly becoming a pandemic: approximately 11% of men and 15% of women, and more than 500 million adults worldwide, now have obesity [5,6]. According to the National Institutes of Health, obesity is considered to be present if an individual’s body mass in kilograms, divided by their height in meters, squared (the body mass index), is >30 kg/m^2^ [7,8]. Numerous studies have shown that the metabolic dysfunction caused by obesity is exacerbated by environmental changes, such as lifestyle, dietary habits, and COVID-19 [9].

Obesity is caused by the accumulation of excess lipid in adipose tissue, and the consequent expansion of adipose tissue involves both the hypertrophy and hyperplasia of adipocytes [10,11]. Adipose tissue is the largest energy storage organ in the body [12], and it stores this energy is the form of triglyceride (TG) in lipid droplets within adipocytes [13,14]. The enlargement of these lipid droplets and the increase in adipose tissue mass ultimately cause metabolic defects, including insulin resistance [15,16]. Lipolysis is the metabolic process by which accumulated fat in adipose tissue breaks down into free fatty acids, which is related to the production of energy [17]. Hormone-sensitive lipase (HSL) is a key enzyme used in the lipolysis process, mediating triglyceride hydrolysis to provide free fatty acids and glycerol in adipocytes [18]. In addition, adipogenesis is the process by which adipocyte precursor cells proliferate and differentiate into mature adipocytes [19]. It is an important process that determines the number of adipocytes, and is mediated by transcription factors such as CCAAT/enhancer-binding protein alpha (CEBPα) and peroxisome proliferator-activated receptor gamma (PPARγ) [20,21]. Therefore, in order to effectively treat obesity, it may be effective to find substances that promote lipolysis and inhibit adipogenesis.

*Lagerstroemia speciosa*, also called “banaba”, is native to tropical southeast Asia, including the Philippines, Vietnam, Malaysia, and southern China [22,23]. It is a tree that can grow as tall as 20 m and has oval leaves. Banaba leaf extracts have been used for many years in folk medicine for the treatment of diabetes [14]. Interestingly, although the first studies of the insulin-like hypoglycemic effects of banaba were conducted in the early 1940s, it was not until 50 years later that scientific interest regarding the effect of banaba to prevent diabetes began to increase again [24]. Numerous studies have focused on the role of corosolic acid in banaba leaf extracts, because it is one of the most active ingredients of banaba. It is a triterpene acid (2α-hydroxyursolic acid) that has been reported to have significant effects on insulin sensitivity and glucose uptake [25]. In addition, a recent study showed that *L. speciosa* extracts inhibit adipogenesis and lipogenesis in 3T3-L1 adipocytes [24]. However, there have been no in vivo studies of the anti-obesity effects of *L. speciosa* leaf extracts.

Probiotics, including *Lactobacillus* species, are beneficial for the host when their diet is supplemented in adequate quantities [26,27]. *Lactobacillus* is a facultative anaerobic bacterium of the phylum Firmicutes that is rod-shaped, gram-positive, and non-spore-forming [28]. These bacteria are also referred to as lactic acid-producing bacteria (LAB), because they metabolize carbohydrates to produce lactic acid [28,29]. Numerous studies are being conducted on gut microbiota and dietary supplementation with a mixture of *Lactobacillus* HY7601 and KY1032. In our previous study, *Lactobacillus curvatus* HY7601 (HY7601) and *Lactobacillus plantarum* KY1032 (KY1032) were demonstrated to have substantial anti-obesity effects in vitro and in vivo [30,31,32]. Specifically, this *Lactobacillus* mixture has been reported to improve the lipid metabolism of high-fructose diet-fed mice and the 3T3-L1 cell line [33]. In addition, we have reported that HY7601 and KY1032 probiotics have anti-obesity effects via a modification of the composition of the gut microbiota in a 12-week human clinical trial: they not only increased the abundances of Bifidobacteriaceae and Akkermansiaceae, but also reduced the abundance of Prevotellaceae and Selenomonadaceae [34]. However, it has not yet been determined whether a mixture of *Lactobacillus* HY7601 and KY1032 might have an additive effect when consumed in combination with an *L. speciosa* extract. Therefore, we evaluated the anti-obesity effect of *Lactobacillus* HY7601 and KY1032 administered to high-fat diet (HFD)-fed obese mice in combination with *L. speciosa* leaf extract.

## 2. Materials and Methods

### 2.1. Preparation of Lactobacillus Mixtures and Banaba Leaf Extract

*Lactobacillus* mixtures were prepared that were composed of KY1032 and HY7601 isolated from kimchi and provided by hy Co., Ltd. (Yongin-si, Republic of Korea) [30]. KY1032 and HY7601 were cultured in De Man–Rogosa–Sharpe (MRS) broth (BD Difco, Sparks, MD, USA) at 37 °C for 24 h, then washed using sterile PBS to remove the medium. KY1032 and HY7601 were then freeze-dried, and the number of colony-forming units (CFU)/g in each viable powdered bacterial preparation were measured. *Lactobacillus* mixtures were made by mixing KY1032 and HY7601 in a 1:1 ratio, based on the colony-forming unit (CFU) of the preparations. Banaba leaf extract containing 1% (*v*/*v*) corosolic acid was purchased from Sigma-Aldrich (St. Louis, MO, USA).

### 2.2. Animal Experiment

Male 6-week-old C57Bl/6 mice were procured from Doo-Yeol Biotech (Seoul, Republic of Korea) and maintained in a controlled temperature and humidity environment (22 ± 1 °C and 55% ± 10%) under a 12-h light/dark cycle. After the mice had adapted to their environment over 1 week, they were randomized to the following treatments for 5 weeks (*n* = 7): control mice fed normal chow diet (CON, AIN-93G), high-fat diet (HFD)-induced obese mice (HFD, 60% kcal as fat), HFD-induced obese mice administrated with the *Lactobacillus* mixture (HFD + L 10^9^ CFU/kg/day), HFD-induced obese mice administrated with the banaba leaf extract containing 1% corsolic acid (HFD + BN, 27 mg/kg/day), and HFD-induced obese mice administrated with 10^9^ CFU/kg/day of the *Lactobacillus* mixture and 27 mg/kg/day of banaba leaf extract (HFD + LBN). The *Lactobacillus* mixture and the banaba leaf extract were dissolved in sterile PBS and orally administered to the mice, and the mice in the CON and HFD groups were orally administered an equal volume of the vehicle. The animal procedures were approved by the Institutional Animal Care and Use Committee of hy Co., Ltd. (IACUC approval number AEC-2024-0004-Y).

### 2.3. Measurement of Body Mass and Tissue Wieght

During the study, the body mass and food and water consumption of the mice were measured once weekly. At the end of the treatment period, the mice were starved overnight and then euthanized using carbon dioxide (CO_2_). The inguinal, epididymal, and brown fat depots and the livers and spleens of mice were harvested. The masses of the collected samples were measured, and then the inguinal fat, epididymal fat, and liver samples were frozen and stored at −80 °C for subsequent gene expression analysis.

### 2.4. Analysis of Circulating Biochemical Parameters

Blood samples were also collected, and serum samples were prepared for biochemical analyses. Blood samples were centrifuged at 3000× *g* for 20 min to separate the serum. The serum triglyceride (TG, MBS726589), total cholesterol (T-Chol, MBS269999), high-density lipoprotein-cholesterol (HDL-Chol, MBS763033), low-density lipoprotein-cholesterol (LDL-Chol, MBS706909), glucose (MBS7200879), and insulin (ab277390) concentrations; the HbA1c level (MBS2024955); and the lactate dehydrogenase (LDH, MBS260522), creatinine kinase (ab285231), aspartate aminotransferase (AST, ab263882), and alanine aminotransferase (ALT, ab282882) activities were determined using commercial Enzyme-Linked Immunosorbent Assay (ELISA) kits. The absorbance of the samples was measured at the appropriate wavelengths using a microplate reader (Biotek Instruments Inc., Winooski, VT, USA).

### 2.5. Histologic Analysis of Tissues

Samples of inguinal and epididymal fat depots were fixed in 10% neutral-buffered formalin solution at 24 h, then embedded in paraffin blocks, sectioned, and stained with hematoxylin and eosin (H&E). The slides were examined under a Zeiss Axiovert 200M microscope (Carl Zeiss AG, Oberkochen, Germany), and the number of adipocytes per unit area were counted on slides of inguinal and epididymal adipose tissue. In addition, the horizontal and vertical diameters of the central lipid droplets were measured using Image J software version 1.53g (NIH, Bethesda, MD, USA) and the mean diameters were calculated. All the measurements were repeated at least six times, and the mean values were calculated.

### 2.6. RNA Isolation, cDNA Synthesis, and Gene Expression Analysis

RNA was extracted from inguinal fat, epididymal fat, and liver samples using an Easy-spin RNA Extraction Kit (iNtRON Biotechnology, Gyeonggi-do, Republic of Korea) and an easy-BLUE^TM^ Solution lysis buffer. cDNA synthesis was performed using Maxime RT PreMix (LiliF^TM^ Diagnostics, Seoul, Republic of Korea) at 45 °C for 60 min, followed by deactivation at 95 °C for 5 min. The concentrations of the synthesized cDNA were quantified using NanoDrop 2000 (Thermo Scientific, Waltham, MA, USA). Gene expression analysis was conducted on a QuantStudio 6-Flex Real-time PCR System (Applied Biosystems, Foster City, CA, USA) using Gene Expression Master Mix (Applied Biosystems) and mouse-specific TaqMan probes (Applied Biosystems). The TaqMan probes used were as follows: glyceraldehyde-3-phosphate dehydrogenase (*Gapdh*, Mm99999915_g1), CCAAT enhancer-binding protein alpha (*Cebpα*, Mm00514283_s1), fatty acid synthase (*Fas*, Mm00662319_m1), fatty acid binding protein 4 (*Fabp4*, Mm00445878_m1), peroxisome proliferator-activated receptor gamma (*Pparγ*, Mm00440945_m1), *hormone sensitive* (*HSL*, Mm00495359_m1), *Forkhead box protein O1* (*foxo1*, Mm00490671_m1), and adiponectin (*Adipoq*, Mm04933656_m1). *Gapdh* was used as the reference gene and to normalize target gene expression, which was calculated using the 2^−∆∆CT^ method.

### 2.7. Statistical Analysis

Statistical analysis was performed using SPSS version 12.0 (IBM, Inc., Armonk, NY, USA). Data are expressed as the mean ± standard deviation and datasets were compared using one-way ANOVA, followed by Duncan’s test. *p* < 0.05 was considered to indicate statistical significance.

## 3. Results

### 3.1. The Consumption of Lactobacillus Mixture and the L. speciosa Leaf Extract Reduces the Body Weight of HFD-Fed Obese Mice

**Figure 1:**
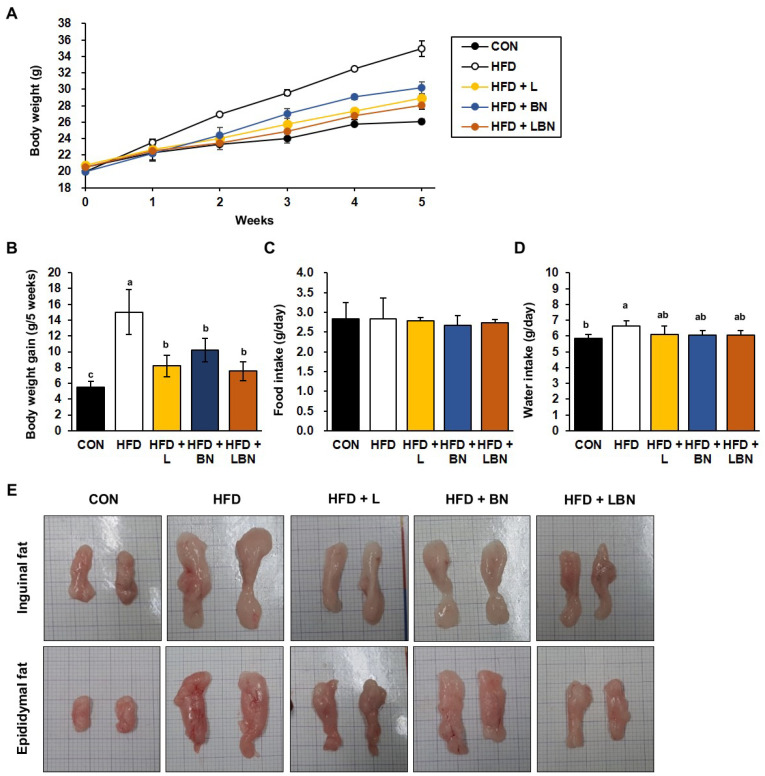
Effects of *Lactobacillus* mixture and banaba leaf extract on the body masses of high-fat diet-fed mice. (**A**) Body masses of the mice each week. (**B**) Body mass gain for experimental duration. (**C**) Food intake per day. (**D**) Water intake per day. (**E**) Macroscopic images of the inguinal and epididymal fat depots. CON, chow diet-fed control mice; HFD, high-fat diet-fed obese mice; HFD + L, HFD-fed mice treated with 10^8^ CFU/kg/day of *Lactobacillus* HY7601 and KY1032; HFD + B, HFD-fed mice treated with 27 mg/kg/day of banaba leaf extract; HFD + LBN, HFD-fed mice treated with 10^8^ CFU/kg/day of the *Lactobacillus* mixture and 27 mg/kg/day of the banaba leaf extract. Data are mean ± SD. Different letters indicate significant differences (*p* < 0.05): a > ab > b > c.

The HFD group had a higher body mass from week 2 of the study, as shown in Figure 1A. After 5 weeks, the mean body mass of the HFD group (34.96 ± 0.93 g) was significantly higher than that of the CON group (26.09 ± 0.30 g), indicating that the HFD group had induced obesity. However, the final mean body masses of the HFD + L and HFD + BN groups were significantly lower (28.93 ± 0.43 g and 30.21 ± 0.73 g, respectively). In addition, the body masses of the HFD + LBN group were significantly lower than those of the HFD group (28.07 ± 0.52 g). Similarly, the body mass gains of the CON and HFD mice were 5.54 g and 15.01 g for 5 weeks, respectively (Figure 1B), and those of the three groups treated with the experimental preparations were lower than those of the HFD group. The mass gain of the HFD + LBN group was the lowest of these three (8.19 g for HFD + L, 10.22 g for HFD + BN, and 7.53 g for HFD + LBN for 5 weeks). However, there were no significant differences in the food intakes of the five groups. By contrast, the water intake of the HFD mice was significantly higher than that of the CON mice, but those of HFD + L, HFD + BN, and HFD + LBN groups were lower. These findings imply that treatment with a combination of the probiotic mixture (KY1032 and HY7601) and *L. speciosa* leaf extract has an additive effect in inhibiting weight gain, compared with the oral administration of the two on their own. Lastly, as shown in Figure 1E, both depots of inguinal fat and epididymal fat were larger after 5 weeks in the HFD-fed mice than in the CON mice. However, the administration of the *Lactobacillus* mixture, the banaba leaf extract, or both together reduced the sizes of these depots.

### 3.2. The Consumption of Lactobacillus Mixture and L. speciosa Leaf Extract Suppresses the Adipose Tissue Expansion of HFD-Fed Obese Mice

After 5 weeks of HFD consumption, the inguinal and epididymal adipose tissue depots were significantly larger in the HFD group (by 289% and 345%, respectively) than in the CON group. However, the masses of the *Lactobacillus* mixture-treated group were significantly lower than those of the HFD group (by 58% and 58%, respectively), as were those of the banaba leaf extract-treated mice (by 56% and 66%, respectively). And those of the mice fed a combination of these were the lowest at 45% and 55%, respectively. However, there were no differences in the masses of the brown fat depot, liver, or spleen among the groups (Table 1). Thus, there was an additive effect of the combination of the *Lactobacillus* mixture and the banaba leaf extract on the white adipose tissue masses of HFD-fed obese mice.

### 3.3. The Lactobacillus Mixture and the L. speciosa Leaf Extract Alter the Serum Lipid Concentrations of HFD-Fed Obese Mice

The serum TG, T-Chol, HDL-Chol, and LDL-Chol concentrations of the HFD mice were significantly higher than those of the CON mice. After 5 weeks of administration of the test preparations, the HFD + L group had significantly lower serum TG, T-Chol, and LDL-Chol concentrations than those of the HFD mice; and the HFD + BN group had significantly lower T-Chol and LDL-Chol concentrations than the HFD mice. Furthermore, the administration of the combination reduced the serum TG and T-Chol concentrations more than the individual substances. Specifically, the serum LDL-Chol concentration of the HFD + LBN group was slightly lower than that of the HFD + BN group, but not significantly. But it was significantly lower than the serum LDL-Chol concentration of the HFD + L group. By contrast, HFD feeding significantly increased the HDL-Chol concentrations of the mice, but none of the treatments affected this parameter (see Table 2).

### 3.4. The Lactobacillus Mixture and the Banaba Leaf Extract Reduce the Inguinal and Epididymal Fat Depot Masses of HFD-Fed Obese Mice

**Figure 2:**
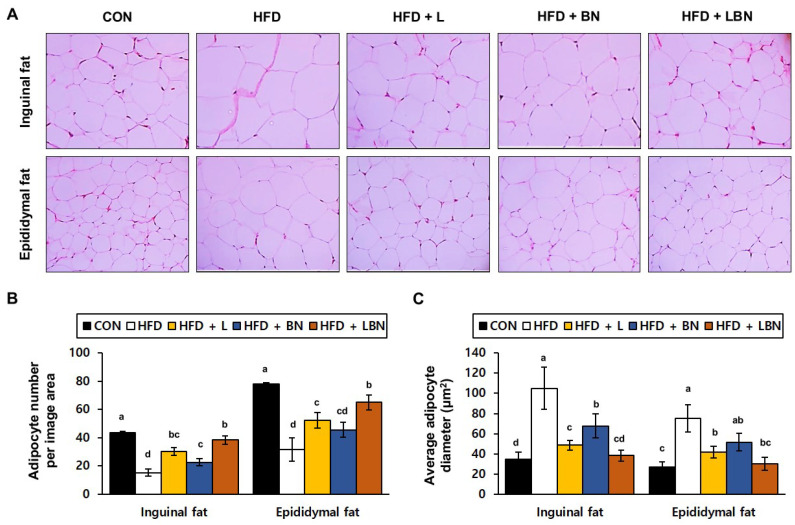
Effects of the *Lactobacillus* mixture and the banaba leaf extract on the adipose depts of the mice. (**A**) Hematoxylin and eosin (H&E)-stained histologic images of the depots. (**B**) Number of adipocytes per unit area. (**C**) Adipocyte diameter. Con, chow diet-fed control mice; HFD, high-fat diet-fed mice; HFD + L, HFD-fed mice treated with 10^8^ CFU/kg/day of HY7601 and KY1032; HFD + B, HFD-fed mice treated with 27 mg/kg/day of banaba leaf extract; HFD + LBN, HFD-fed mice treated with 10^8^ CFU/kg/day of the *Lactobacillus* mixture and 27 mg/kg/day of the banaba leaf extract. Data are mean ± SD. Different letters indicate significant differences (*p* < 0.05): a > ab > b > bc > c > cd > d.

To determine the effects of the *Lactobacillus* mixture and the banaba leaf extract on the adipose tissue of the HFD-fed mice, we evaluated the histology of the adipocytes in the inguinal and epididymal fat depots. A recent study showed that epididymal fat is characterized by a higher degree of vascularization than inguinal fat. We found significant differences in both the inguinal and epididymal fat depots between the control and HFD groups. We measured the sizes of the adipocytes and counted them on histologic sections of each depot. As shown in Figure 2C, there were fewer adipocytes per unit area in the inguinal and epididymal fat depots of the HFD mice (15.3 ± 2.52 and 31.7 ± 8.14 adipocytes/μm^2^, respectively) than in those of the CON mice (43.67 ± 6.66 and 78.00 ± 9.54 adipocytes/μm^2^, respectively). However, the banaba leaf extract (30.33 ± 2.89 and 52.33 ± 5.51 adipocytes number/μm^2^, respectively), the *Lactobacillus* mixture (22.67 ± 2.52 and 45.67 ± 5.13 adipocytes/μm^2^, respectively), and the two together (38.33 ± 3.06 and 65.00 ± 5.29 adipocytes/μm^2^, respectively), significantly increased the number of adipocytes per unit area in the HFD-fed mice. The larger number of adipocytes in the HFD mice might suggest that fat accumulation and differentiation into mature adipocytes were suppressed. Finally, as shown in Figure 2C, the mean adipocyte diameters of the HFD mice (105.08 ± 20.63 μm and 75.22 ± 13.63 μm in the inguinal and epididymal fat depots, respectively) were much higher than those of the CON mice (34.55 ± 7.00 μm and 27.24 ± 4.86 μm, respectively). However, the adipocytes of the HFD + L (48.62 ± 4.87 μm and 41.54 ± 5.73 μm), HFD + BN (67.68 ± 11.81 μm and 51.68 ± 8.61 μm), and HFD + LBN (38.26 ± 5.66 μm and 30.33 ± 6.60 μm) groups were smaller than those of the HFD group.

### 3.5. The Lactobacillus Mixture and the Banaba Leaf Extract Reduce the Hyperglycemia of the HFD-Fed Mice

**Figure 3:**
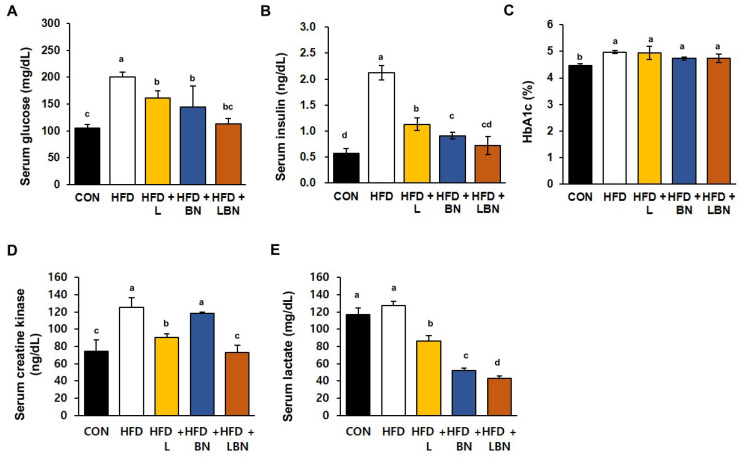
Effects of the *Lactobacillus* mixture and the banaba leaf extract on serum parameters. (**A**) Serum glucose concentration. (**B**) Serum insulin concentration. (**C**) HbA1c level. (**D**) Creatine kinase activity. (**E**) Lactate concentration. Con, chow diet-fed control mice; HFD, high-fat diet-fed obese mice; HFD + L, HFD mice treated with 10^8^ CFU/kg/day of HY7601 and KY1032; HFD + B, HFD mice treated with 27 mg/kg/day of banaba leaf extract; HFD + LBN, HFD mice treated with 10^8^ CFU/kg/day of *Lactobacillus* and 27 mg/kg/day of banaba leaf extract. Data are mean ± SD. Different letters indicate significant differences (*p* < 0.05): a > b > bc > c > cd > d.

The serum glucose and insulin concentrations and the HbA1c level significantly differed between the HFD and CON groups, as shown in Figure 3A–C. Specifically, the mean serum glucose concentration of the HFD mice was 190% higher than that of the CON mice. However, treatment with the *Lactobacillus* mixture or the banaba leaf extract reduced the mean glucose concentration of the HFD mice by 80.7% and 72.3%, respectively, and the two together reduced this concentration by 56.7%. In addition, the mean serum insulin concentration of the HFD group was 373% higher than that of the CON group. However, the *Lactobacillus* mixture and the banaba leaf extract significantly reduced this concentration, by 53.3% and 42.8%, respectively. In addition, the HFD mice administered with both treatments showed a significantly larger reduction in insulin concentration than those administered with either preparation alone (Figure 3B). Furthermore, the HbA1c level of the HFD mice (4.97 ± 0.06%) was significantly higher than that of the CON mice (4.47 ± 0.06%), but the levels of the HFD + L, HFD + BN, and HFD + LBN groups did not differ from those of the former group. These data indicate that supplementation with the *Lactobacillus* mixture and/or the banaba leaf extract reduces the fasting blood glucose concentrations of HFD-fed obese mice. We also measured the serum creatine kinase activities and lactate concentrations of the mice, both of which are associated with insulin resistance. The mean serum creatine kinase activity of the HFD mice was 168% higher than that of the CON mice. And this was reduced more effectively by *Lactobacillus* treatment (72.3%) than by banaba leaf extract treatment (94.7%), but the reduction was larger when the two were administered together (58.5%), compared to HFD. In contrast, the HFD diet did not affect the mean serum lactate concentration of the mice, but it was significantly reduced by the *Lactobacillus* mixture (67.5% vs. HFD mice), the banaba leaf extract (41.1% vs. HFD mice), and the combination of the two (33.7% vs. HFD mice), compared to HFD.

### 3.6. The Lactobacillus Mixture and the Banaba Leaf Extract Alters the Expression of Adipogenic Genes in HFD-Fed Obese Mice

**Figure 4:**
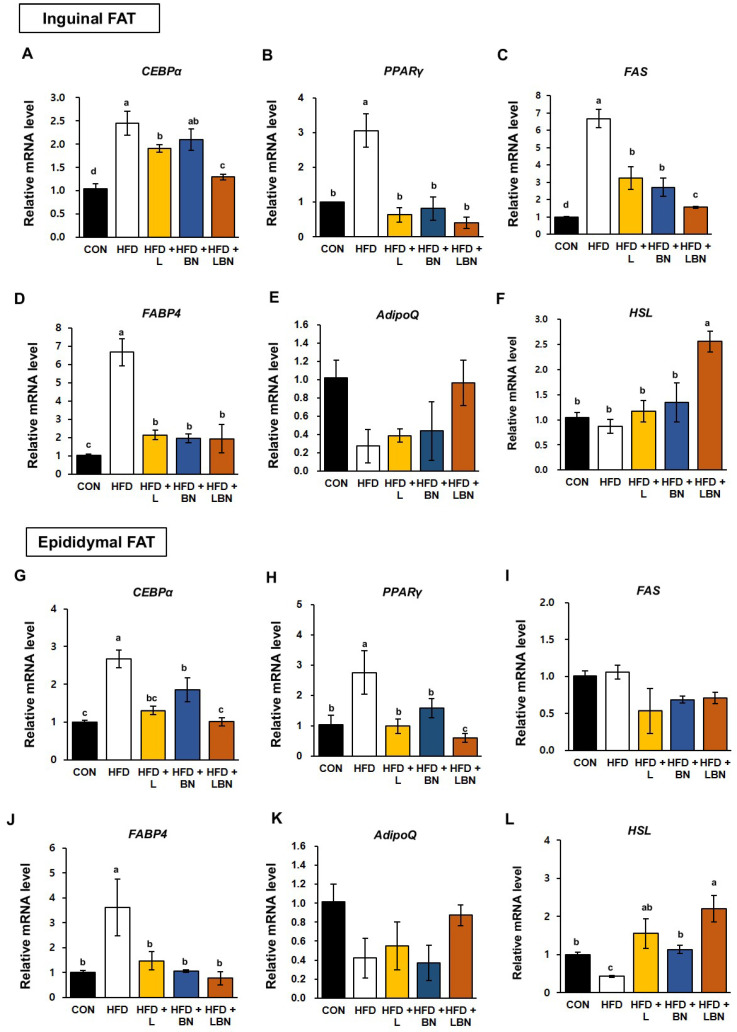
Effects of the *Lactobacillus* mixture and the banaba leaf extract on the expression of adipogenic genes in white adipose tissue. The relative mRNA expression of (**A**) *CEBPα*, (**B**) *PPARγ*, (**C**) *FAS*, (**D**) *FABP4*, (**E**) *AdipoQ,* and (**F**) HSL in inguinal fat are shown, as is the relative mRNA expression of (**G**) *CEBPα*, (**H**) *PPARγ*, (**I**) *FAS*, (**J**) *FABP4*, (**K**) *AdipoQ* and (**L**) HSL in epididymal fat. Con, chow diet-fed control mice; HFD, high-fat diet-fed obese mice; HFD + L, HFD mice treated with 10^8^ CFU/kg/day of HY7601 and KY1032; HFD + B, HFD mice treated with 27 mg/kg/day of banaba leaf extract; HFD + LBN, HFD mice treated with 10^8^ CFU/kg/day of the *Lactobacillus* mixture and 27 mg/kg/day of the banaba leaf extract. Data are mean ± SD. Different letters indicate significant differences (*p* < 0.05): a > ab > b > bc > c > d.

White adipose tissue depots, such as the inguinal and epididymal depots of rodents, are responsible for lipid storage [35,36]. To better understand the molecular mechanisms of the effects of the *Lactobacillus* mixture and the banaba leaf extract on lipid accumulation and adipogenesis, we measured the mRNA expression of the genes encoding hormone-sensitive lipase (HSL), CCAAT/enhancer-binding protein alpha (*CEBPα*), peroxisome proliferator-activated receptor gamma (*PPARγ*), fatty acid synthase (*FAS*), fatty acid-binding protein *4* (*FABP4*), and adiponectin (*AdipoQ*) in the two white adipose tissue depots. As shown in Figure 4A–D, the mRNA expression of *CEBPα*, *PPARγ*, *FAS*, and *FABP4* was significantly higher in the inguinal fat depot of the HFD-fed mice (by 2.45-fold, 2.76-fold, 6.68-fold, and 6.70-fold, respectively) than in that of the control mice. However, the administration of the *Lactobacillus* mixture significantly reduced the mRNA expression of these genes (1.91-fold, 0.99-fold, 3.24-fold, and 2.15-fold, respectively) in HFD mice. The mRNA expression of *CEBPα*, *PPARγ*, *FAS*, and *FABP4* was also reduced by the banaba leaf extract (2.10-fold, 1.58-fold, 2.72-fold, and 1.95-fold, respectively), but there was no significant difference in the expression of *CEBPα*. Moreover, when the HFD mice were treated with the combination, the mRNA expression of *CEBPα*, *PPARγ*, *FAS*, and *FABP4* was 1.29-fold, 0.60-fold, 1.56-fold, and 1.94-fold lower, respectively.

In addition, as shown in Figure 4G–J, the mRNA expression of *CEBPα*, *PPARγ*, and *FABP4* in epididymal fat was higher in the HFD group than in the CON group (by 2.67-fold, 3.06-fold, and 3.62-fold, respectively). However, the mRNA expression of *CEBPα*, *PPARγ*, and *FABP4* in the epididymal fat depot was reduced by the *Lactobacillus* mixture (1.31-fold, 0.63-fold, and 1.47-fold, respectively) and by the banaba leaf extract (1.85-fold, 0.82-fold, and 1.06-fold, respectively). The administration of both treatments reduced the mRNA expression of *CEBPα*, *PPARγ*, and *FABP4* by 0.71-fold, 0.40-fold, and 0.76-fold, respectively. By contrast, the *FAS* expression in the epididymal fat depot of the HFD mice did not differ significantly from that of the CON mice, but was significantly reduced by the three treatments. Also, the mRNA expression of *Adiponection (AdipoQ)* in both white adipose tissue depots was slightly, but not significantly, lower in the HFD group than in the CON group, and when the two treatments were administered together, the expression of *AdipoQ* tended to be restored to that of the CON group. Finally, the mRNA level of the lipolysis related gene (*Hormone sensitive lipase, HSL*) was significantly highest in the HFD group treated with the combination of *Lactobacillus* mixture and the banaba leaf extract.

### 3.7. The Lactobacillus Mixture and the Banaba Leaf Extract Reduce Liver Enzyme Activities and Reduce the Hepatic Expression of Lipogenic Genes in HFD-Fed Obese Mice

**Figure 5:**
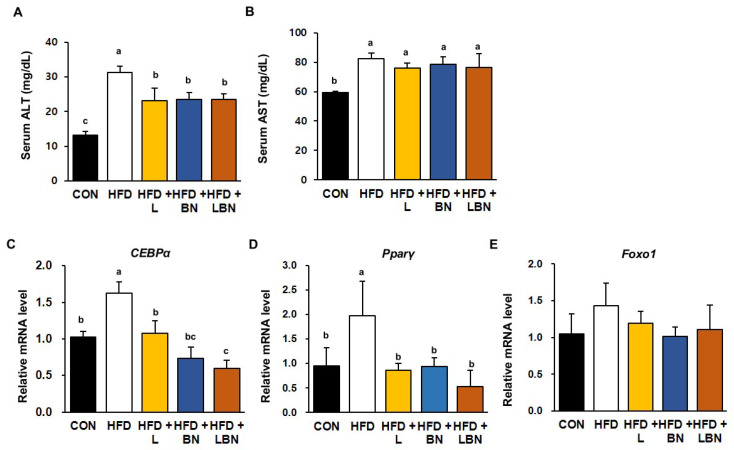
Effects of the *Lactobacillus* mixture and the banaba leaf extract on serum liver enzyme activities and hepatic adipogenic gene expression. Serum activities of (**A**) ALT and (**B**) AST. Relative hepatic mRNA expression of (**C**) *CEBPα* and (**D**) *PPARγ* (**E**) *Forkhead box protein O1* (*Foxo1*). Con, chow diet-fed control mice; HFD, high-fat diet-fed obese mice; HFD + L, HFD mice treated with 10^8^ CFU/kg/day of HY7601 and KY1032; HFD + B, HFD mice treated with 27 mg/kg/day of banaba leaf extract; HFD + LBN, HFD mice treated with 10^8^ CFU/kg/day of the *Lactobacillus* mixture and 27 mg/kg/day of the banaba leaf extract. Data are mean ± SD. Different letters indicate significant differences (*p* < 0.05): a > b > bc > c.

To characterize the effects of the *Lactobacillus* mixture and the banaba leaf extract on the livers of HFD-fed obese mice, we first measured the serum activities of liver enzymes (Figure 5A,B). In mice, 5 weeks of HFD feeding significantly increased the serum ALT and AST activities of the mice (ALT: 31.3 ± 1.7 mg/dL vs. 13.20 ± 1.06 mg/dL and AST: 82.3 ± 3.7 mg/dL vs. 59.3 ± 0.83 mg/dL for the HFD and control groups, respectively). The serum ALT and AST activities were lower in all the treatment groups than in the HFD group, but there was no additive effect of the *Lactobacillus* mixture and the banaba leaf extract. We also measured the mRNA expression of *CEBPα* and *PPARγ* in the livers of the HFD-fed obese mice and found that HFD feeding increased the expression of *CEBPα* (by 1.63-fold) and *PPARγ* (by 1.98-fold), but that the administration of the *Lactobacillus* mixture and the banaba leaf extract restored this to the level of the CON mice. Furthermore, when the combination was administered, the expression of *CEBPα* and *PPARγ* were reduced by 0.60-fold and 0.52-fold, respectively. However, as shown in Figure 5E, the mRNA level of Foxo1, a fatty acid oxidation-associated gene, did not show a significant difference among all groups. Thus, the combination of the *Lactobacillus* probiotics and the banaba leaf extract might have partially beneficial effects on lipid metabolism in the livers of HFD-fed obese mice.

## 4. Discussion

Obesity is an increasingly important global public health problem that is caused by imbalances in the diet and a lack of exercise [37]. Obesity is characterized by a significant increase in body fat mass [38] and is closely associated with dyslipidemia, type 2 diabetes mellitus, oxidative stress, chronic inflammation, heart disease, hypertension, fatty liver disease, and a variety of cancers [39]. Despite the discovery of drugs that show promise for the treatment of obesity, these often have side effects and associated safety concerns [40,41]. Various strains of lactic acid-producing bacteria have been reported to help individuals achieve weight loss safely, and the mechanism involves gut–brain communication on a molecular level [42,43]. In our previous study, we found that a mixture of *Lactobacillus curvatus* HY7601 and *Lactobacillus plantarum* KY1032 not only ameliorates the defects in lipid metabolism that characterize rats fed a high-fructose diet, but also limits visceral fat deposition in humans [32]. In addition, this *Lactobacillus* mixture ameliorates overweight by altering the gut microbial composition and beta diversity in humans. However, there have been no studies of whether this mixture retains its anti-obesity effects or has additive effects to those of other natural substances.

The treatment of obesity and insulin resistance using natural products, including various plant-derived materials, has recently been attracting attention [44,45]. Numerous studies have shown that the drugs used for the treatment of hyperglycemia may cause a deterioration of the overweight or obesity that is one of the principal causes of type 2 diabetes [46,47]. Therefore, to identify new antidiabetic drugs that are hypoglycemic but do not exacerbate adiposity, various herbal materials have been evaluated for use in combination treatments [48]. Among these, the leaves of *L. speciosa* (Lythraceae), also known as banaba, have been traditionally used to treat type 2 diabetes-related disorders, inflammation, and apoptosis [25,49]. The banaba leaf extract that we used in the present study contained 1% (*v*/*v*) corosolic acid (2α-hydroxyursolic acid), according to HPLC analysis. In a recent study, the oral administration of corosolic acid was shown to reduce the insulin resistance and adiposity of mice [48]. According to a recent paper, corosolic acid was reported to improve lipid metabolism and serum insulin concentration by improving Th17 inflammation [50,51]. Therefore, we aimed to evaluate the anti-obesity effects of *Lactobacillus* mixture (HY7601 and KY1032) and banaba leaf extract (*L. speciose*) in HFD-fed obese mice in the present study. To this end, we administered the same concentrations of both substances to the experimental groups and compared the anti-obesity effects of each preparation on its own and in combination.

After 5 weeks of the study, the body mass of the HFD group was significantly higher than that of the CON group, but the *Lactobacillus* mixture reduced the body mass gain of the HFD-fed mice. This effect was larger than that of the banaba leaf extract, but the combination of the two caused a larger reduction in body mass gain. However, whether the preparations were administered alone or in combination, the daily food intake of the mice was not affected. Therefore, both preparations can be treated as promising dietary supplements that suppress high-fat diet induced weight gain without affecting food intake. White adipose tissue is a key site for energy storage [52]. During periods of excessive food intake, energy is stored in the form of TG, which causes an increase in visceral fat mass [2,53]. This increase is closely associated with hypercholesterolemia, hyperlipidemia, insulin resistance, and a higher risk of cardiovascular disease [54,55,56]. In the present study, the *Lactobacillus* mixture and the banaba leaf extract both significantly reduced the sizes of the inguinal and epididymal fat depots of the HFD-fed mice. In particular, the size of the inguinal fat depot was reduced more effectively by each preparation alone, rather than in combination. However, there were no significant differences in the masses of the brown fat depot, liver, or spleen among the experimental groups. Thus, the lactic acid-producing bacteria and the banaba leaf extract specifically affect the mass of white adipose tissue. We also performed histologic analyses of the two white adipose depots, and found that the central lipid droplets of the HFD mice were smaller than those of the CON mice and that the number of adipocytes per unit area was significantly increased, consistent with the magnitude of the effect, by the banaba leaf extract, *Lactobacillus* mixture, and both together in the mice. Furthermore, Figure 2C shows that the adipocytes of the HFD mice were much larger than those of the CON mice. These data imply that adipocyte differentiation was induced by HFD feeding. However, the central lipid droplets were significantly reduced in size, in order of the magnitude of the effect, by the *Lactobacillus* mixture, the banaba leaf extract, and the combination of the two. Because the size of the central lipid droplets was increased by HFD feeding, obesity significantly reduced the number of adipocytes per observed area. In addition, the number of adipocytes per unit area was significantly higher in the HFD + L, HFD + BN, and HFD + LBN groups than in the HFD group. White adipose tissue, including the inguinal and epididymal depots, secretes a variety of cytokines that regulate metabolism. In particular, adiponectin is secreted by fat tissue and its circulating concentration is lower in animals and humans with obesity. Adiponectin reduces the plasma TG concentration and improves glucose metabolism by increasing insulin sensitivity. In the present study, we have shown that the expression of the gene encoding adiponectin was higher in both white adipose depots when the *Lactobacillus* mixture and the banaba leaf extract were administered together, rather than separately.

We also measured the serum concentrations of TG, T-Chol, LDL-Chol, and HDL-Chol, and found that the combination treatment caused the largest changes of the three. Specifically, this combination caused significant decreases in TG and T-Chol vs. the HFD, HFD + L, and HFD + BN groups. However, although the HDL-Chol concentration was significantly reduced by HFD feeding, there were no effects of the experimental preparations. Thus, the *Lactobacillus* mixture and the leaf extract of *L. speciosa* ameliorate the dyslipidemia of HFD-fed mice.

Adipose tissue plays a vital role in the regulation of energy supply and storage [57], and adipogenesis is the important process by which preadipocytes differentiate into lipid-rich adipocytes [58]. This process is controlled by a complex, highly orchestrated gene expression program, and involves the accumulation of lipid in the droplets within white adipocytes [58,59]. According to the findings of numerous previous studies, C/EBPα and PPARγ are key regulators of adipogenesis, and their expression leads to the differentiation of preadipocytes into mature adipocytes [60,61,62]. In many recent studies, researchers have attempted to prevent obesity by altering the expression of the transcription factors that regulate adipogenic genes, such as those encoding FAS and FABP4 [63,64]. FAS is an important protein in both lipogenesis and adipogenesis, and recent studies have shown that 3T3-L1 adipocyte differentiation is inhibited by reducing the expression of FAS [65,66]. FABP4, also known as adipocyte protein 2 (aP2), has been reported to be the important transcription factor of adipocytes and to be essential for energy homeostasis [67,68]. FABP4 maintains adipocyte homeostasis and is a regulator of lipolysis and lipogenesis, which involves interactions with PPAR-γ [69,70]. In the present study, we aimed to determine whether a combination of a probiotic mixture (HY7601 and KY1032) and leaf extract of *L. speciosa* would reduce fat accumulation in the white fat depots of HFD-fed obese mice. Therefore, we measured the mRNA expression of the adipogenic genes *PPARγ*, *CEBPα*, *FAS*, and *FABP4*, and found that the expression of these genes was significantly lower in the inguinal and epididymal white adipose depots of the mice. When the two preparations were administered together at the same concentrations, the effects on adipogenic gene expression were significantly larger than when each was administered alone, implying an additive effect. However, the combination reduced the expression of lipogenic genes to a lesser extent than each preparation alone.

High serum activities of liver enzymes, such as ALT and AST, have been reported to be present in several diseases, and high activities have frequently been demonstrated in obese mammals [71,72]. In the present study, we measured the activities of biomarkers, including ALT and AST, to assess the level of liver damage present. Five weeks of HFD feeding resulted in significantly higher serum ALT and AST activities, suggesting that the HFD had induced liver dysfunction in the mice. However, the serum ALT and AST activities were significantly lower in the HFD + L, HFD + BN, and HFD + LBN groups. These results indicate that the tested probiotics and banaba leaf extract limit the development of HFD-induced lipid accumulation and the associated metabolic dysfunction in the liver. Overall, these results suggest that the two preparations limit liver damage and hepatic lipid accumulation in HFD-fed obese mice.

Visceral fat accumulation in particular is responsible for the complications of obesity [73,74], including insulin resistance and the related diseases [75]. In addition, adipogenesis involves changes in insulin sensitivity, as well as changes in cell morphology [76]. In addition to the effects of the probiotics and banaba leaf extract on obesity, we determined whether the combination could ameliorate insulin resistance. We found that the HFD markedly increased the serum glucose and insulin concentrations of the mice, but that the *Lactobacillus* mixture and the banaba leaf extract ameliorated these effects, implying a reduction in insulin resistance. Creatine kinase, a key enzyme in energy metabolism, is associated with insulin resistance and obesity. In addition, lactate is considered to be an important player in energy homeostasis under anaerobic conditions, and a high lactate concentration is thought to be the result of hypoxia in obese adipocytes. In the present study, the two preparations restored their levels to ones similar to those of the CON group. However, the serum creatine kinase and lactate levels of the HFD + LBN mice were the lowest of the HFD-fed groups.

## 5. Conclusions

We have performed a study to evaluate the anti-obesity effects of *Lactobacillus* mixture (HY7601 and KY1032) and *L. speciosa* leaf extract. We found that a combination of 27 mg/kg/day of *L. speciosa* leaf extract and 10^9^ CFU/kg/day *Lactobacillus* mixture had additive effects to reduce weight gain and adipose tissue expansion in HFD-fed obese mice. In addition, they reduced the size of the central lipid droplets in white adipocytes and the expression of genes involved in adipogenesis/lipogenesis in the inguinal and epididymal fat depots and the liver. These results suggest that a combination of probiotics (HY7601 and KY1032) and *L. speciosa* leaf extract inhibits fat accumulation in vivo, and therefore that they represent potential dietary supplements or functional foods for use in individuals with obesity and metabolic disease.

## Figures and Tables

**Table 1:** Effect of the *Lactobacillus* mixture and the banaba leaf extract on the organ masses of the HFD-fed obese mice after 5 weeks. Data are mean ± SD. Different letters indicate significant differences (*p* < 0.05): a > b > c > d.

Group	Inguinal Fat	Epididymal Fat	Brown Fat	Liver	Spleen
CON	13.05 ± 0.57 ^d^	14.95 ± 2.64 ^c^	27.88 ± 0.41	27.88 ± 0.38	2.24 ± 0.13
HFD	37.75 ± 2.85 ^a^	51.59 ± 1.67 ^a^	26.92 ± 0.41	26.92 ± 0.66	2.13 ± 0.11
HFD + L	21.91 ± 1.24 ^b^	30.09 ± 3.73 ^b^	20.92 ± 0.47	28.38 ± 0.77	2.22 ± 0.15
HFD + BN	21.25 ± 3.12 ^b^	34.16 ± 5.97 ^b^	21.25 ± 0.42	29.79 ± 0.58	2.15 ± 0.20
HFD + LBN	17.15 ± 0.39 ^c^	28.31 ± 0.90 ^b^	18.03 ± 0.39	29.28 ± 0.75	2.22 ± 0.20

**Table 2:** Effects of 5 weeks of administration of the *Lactobacillus* mixture and the banaba leaf extract on the serum lipids of HFD-fed obese mice. Data are mean ± SD. Different letters indicate significant differences (*p* < 0.05): a > ab > b > bc > c > d.

Group	Serum Concentration (mg/mL)			
	Triglyceride	Total Cholesterol	HDL-Cholesterol	LDL-Cholesterol
CON	40.80 ± 9.55 ^c^	136.00 ± 5.66 ^d^	89.97 ± 2.78 ^a^	5.80 ± 0.40 ^c^
HFD	77.20 ± 11.88 ^a^	198.67 ± 20.23 ^a^	80.10 ± 2.30 ^b^	15.75 ± 2.98 ^a^
HFD + L	60.00 ± 4.69 ^b^	164.00 ± 5.29 ^b^	80.35 ± 1.81 ^b^	14.05 ± 0.79 ^ab^
HFD + BN	75.20 ± 11.28 ^a^	153.33 ± 11.02 ^bc^	75.10 ± 4.15 ^b^	10.85 ± 1.73 ^b^
HFD + LBN	53.60 ± 7.40 ^bc^	151.33 ± 4.62 ^c^	75.30 ± 3.36 ^b^	9.30 ± 1.36 ^b^

## Data Availability

All the data are contained within the article.
